# Relationship between N-Terminal Pro-Brain Natriuretic Peptide, Obesity and the Risk of Heart Failure in Middle-Aged German Adults

**DOI:** 10.1371/journal.pone.0113710

**Published:** 2014-11-25

**Authors:** Janine Wirth, Brian Buijsse, Romina di Giuseppe, Andreas Fritsche, Hans W. Hense, Sabine Westphal, Berend Isermann, Heiner Boeing, Cornelia Weikert

**Affiliations:** 1 Research Group of Cardiovascular Epidemiology, Department of Epidemiology, German Institute of Human Nutrition Potsdam-Rehbruecke, Nuthetal, Germany; 2 Department of Epidemiology, German Institute of Human Nutrition Potsdam-Rehbruecke, Nuthetal, Germany; 3 Department of Internal Medicine, Division of Endocrinology, Diabetology, Nephrology, Vascular Disease and Clinical Chemistry, University of Tübingen, Tübingen, Germany; 4 Institute of Epidemiology and Social Medicine, Clinical Epidemiology Unit, University Hospital, Münster, Germany; 5 Department for Clinical Chemistry and Pathobiochemistry, Otto-von-Guericke-University Magdeburg, Magdeburg, Germany; 6 Institute for Social Medicine, Epidemiology and Health Economics, Charité University Medical Center, Berlin, Germany; Dasman Diabetes Institute, Kuwait

## Abstract

***Background*:**

Both high concentrations of N-terminal pro-brain natriuretic peptide (NT-proBNP) and obesity are related to higher heart failure risk. However, inverse relationships between NT-proBNP and obesity have been reported. Therefore, it was investigated whether the association between NT-proBNP and the risk of heart failure differed according to obesity status.

***Methods*:**

A case-cohort study was conducted within the European Prospective Investigation into Cancer and Nutrition (EPIC)-Potsdam, comprising a random sub-cohort (non-cases = 1,150, cases = 13, mean age: 50.5±9.0 years) and heart failure cases outside the sub-cohort (n = 197). Weighted Cox proportional hazards regression was used to examine the association between NT-proBNP and heart failure risk during a mean follow-up time of 8 years. Stratified analyses were performed according to obesity status as defined by body mass index (<30 kg/m^2^ versus ≥30 kg/m^2^).

***Results*:**

Overall, NT-proBNP was associated with higher risk of heart failure after multivariable adjustment (hazard ratio (HR) and 95% confidence interval (CI): 2.56 (1.49–4.41) for the top versus bottom tertile of NT-proBNP, p_trend_:<0.01). In stratified analyses, the shape of association was linear in non-obese and U-shaped in obese participants: HRs (95%CI) from the first to the third tertile of NT-proBNP for non-obese: reference, 1.72 (0.85–3.49), 2.72 (1.42–5.22), and for obese: 3.29 (1.04–10.40), reference, 3.74 (1.52–9.21).

***Conclusions*:**

Although high circulating concentrations of NT-proBNP were positively associated with incident heart failure in the entire sample, the association differed according to obesity status. In obese, an increased risk of heart failure was also observed in those with low NT-proBNP concentrations. If confirmed, this observation warrants further investigation to understand underlying pathophysiological mechanisms.

## Introduction

Heart failure (HF) has experienced a considerable increase in prevalence and contributes to a tremendous public health burden [1]. Despite advances in survival and therapeutic opportunities, HF prognosis remains poor, characterized by high fatality and hospitalization rates [1], [2]. The need for action in the context of preventive measures and improvements in risk prediction is obviously desirable.

Brain natriuretic peptides (BNP) and N-terminal pro brain natriuretic peptides (NT-proBNP) are of promising value in early disease prevention and have received increasing interest as cardiac markers [3]. Both are produced in equimolar amounts by enzymatic cleavage of the precursor hormone pro-brain natriuretic peptide (proBNP). ProBNP in turn is mainly released from ventricular myocytes upon activation of the BNP gene, which is induced by excessive stretching of the ventricles due to volume overload and increased filling pressure [4]. While physiological effects of NT-proBNP are currently unknown, BNP affects the vasculature by vasodilation, increased diuresis/natriuresis, and inhibiting renin and aldosterone production as well as vascular and cardiac myocyte growth [4]. Because of the high sensitivity and specificity [5] regarding HF diagnosis, severity assessment, prognosis and therapy [3], the European Society of Cardiology (ESC) has incorporated the measurement of BNP and NT-proBNP into their *Guidelines for the diagnosis and treatment of acute and chronic heart failure* [6] in 2008.

Besides having diagnostic properties, natriuretic peptides might be of considerable predictive value, as increasing evidence indicated strong positive associations between BNP and NT-proBNP levels and the risk of different cardiovascular endpoints [7]. Recently, prospective studies have consistently shown positive associations between BNP/NT-proBNP concentrations and the risk of incident HF [8]–[11].

Overweight and obesity are strong risk factors for HF [12]. The use of highly sensitive and easily measurable biomarkers would be of great benefit for HF prevention, especially in obese, since sensitivity and specificity of traditional devices (e.g. echocardiogram) might be limited in these individuals [13].

Interestingly, better outcomes in morbidity and mortality have been reported among HF patients with overweight and obesity when compared to HF patients with normal weight - a phenomenon termed as “obesity paradox” [14]. Similarly, contradictions emerged with BNP related peptides: despite increased risk of HF with both high concentrations of BNP/NT-proBNP and with the presence of obesity, cross-sectional studies reported inverse associations between natriuretic peptides and markers of anthropometry in individuals with and without HF compared to those with normal weight [15]–[18]. A “natriuretic peptides deficiency” in obese has been speculated [18]. Hence, the diagnostic/prognostic usefulness of BNP/NT-proBNP has been questioned and body mass indices (BMI)-specific threshold values of natriuretic peptides have been recommended to improve diagnostic sensitivity [15], [16].

As far as known, there are no studies that prospectively investigated whether the relationship between NT-proBNP and HF risk differs between obese and non-obese persons. Therefore, the aim of this study was to examine the longitudinal association between NT-proBNP and HF risk with a special emphasis on obesity. Overall, a positive association was hypothesized, which was expected to be weaker in obese compared to non-obese individuals.

## Materials and Methods

### Ethics statement

The present investigation conforms to the principles outlined in the Declaration of Helsinki. All participants gave their written informed consent, and the Ethics Committee of the Federal State Brandenburg approved all study procedures.

### Study population and design

The *European Prospective Investigation into Cancer and Nutrition* (EPIC)-Potsdam comprises 27,548 participants (16,644 women and 10,904 men) aged 35–65 years in women and 40–65 years in men. The recruitment procedures between 1994 and 1998 included physical examinations (e.g. anthropometric measures and blood drawing), a personal interview and questionnaires on socio-demographic and lifestyle characteristics as well as prevalent diseases [19]. Every two to three years, participants were re-contacted and interviewed by questionnaires about lifestyle and newly diagnosed diseases [20]. In the present analysis, the first four follow-up waves were considered, corresponding to a time frame from August 1994 to December 2008. Response rates so far exceeded 90% at each occasion.

A case-cohort study design was used to investigate the relationship between NT-proBNP and HF risk [21]. With this type of study design, results are expected to be generalizable without the need to measure biomarker levels in the entire cohort. Accordingly, a random sample of 1,294 participants was drawn and added up with all incident HF cases. Some exclusion was required for the current analyses (Figure S1). This involved 29 participants with either a prevalent/not verifiable HF at baseline or without any follow-up information. Further exclusion criteria were missing/inappropriate blood samples (n = 196) and missing information about covariates (n = 20), leading to a final study population of 1,360 participants (sub-cohort: n = 1,163 (including 13 HF cases), HF cases outside the sub-cohort: n = 197).

### Outcome Ascertainment

In the present analyses only medically confirmed HF cases were used. Those were identified by several sources of information: self-report [21], death certificates (diagnosis I50 of International Classification of Diseases, 10th revision (ICD-10) as underlying cause of death), and a link to the hospital information system of the major hospital in the Potsdam area. Additionally, participants with incident myocardial infarction or reported drug use typical for HF treatment have been actively enquired for HF. Attending physicians confirmed the diagnosis with specific validation forms and gave information about performed diagnostic procedures. All confirmed cases then were classified into definite, probable and possible HF according to ESC-Guidelines [22]. If the two required criteria (presence of typical symptoms and objective evidence of cardiac dysfunction by echocardiography) were fulfilled, they were classified as definite. Cases were adjudicated as probable, either if they were not symptomatic but had pathological evidence from echocardiogram, or if they were symptomatic but confirmed by other objective evidence (cardiac catheter, electrocardiogram, chest x-ray). Cases with less provided information were defined as possible and if there was no information at all they were excluded from analysis (n = 1).

### Assessment of Exposure and Covariates

NT-proBNP was measured in 2012 at the Institute of Clinical Chemistry, University of Magdeburg, using a solid-phase, two-site chemiluminescent immunometric assay (IMMULITE 2000 Systems Analyzers, SIEMENS). The mean with-in run coefficient of variation was 5.4% at a concentration of 35.6 pg/ml and 4.1% at 29,7 pg/ml. The overall variation coefficients throughout the analyses were 6.4% and 4.7% at the same sample concentration. The analytic measurement range for NT-proBNP was 20.0–35,000 pg/ml. Concentrations below the lower limit of detection (20 pg/ml) (n = 185, ≙14.8%) were set to 10 pg/ml.

Several other biomarkers (e.g. total cholesterol, high density lipoprotein (HDL) cholesterol, high sensitivity C-reactive protein (hsCRP) and creatinine) had been analysed at the Department of Internal Medicine, University of Tübingen [23] and at the Stichting Huisartsen Laboratorium (Breda, The Netherlands).

At baseline, participants' weight and height were measured without wearing shoes with a precision of 0.1 kg and 0.1 cm, respectively. BMI was calculated by dividing body weight by body height squared (kg/m^2^) to assess general obesity (BMI≥30 kg/m^2^). Waist circumference (WC) was measured at the midway between the lower ribs and the iliac crest and hip circumference over the buttocks. Abdominal obesity was defined in two ways, using WC (men: ≥102 cm, women: ≥88 cm) and waist to hip ratio (WHR) (men:>1, women>0.85) [24].

Prevalent hypertension was defined as systolic blood pressure ≥140 mmHg or diastolic blood pressure ≥90 mmHg or self-reporting of a diagnosis or use of antihypertensive medication. Participants were classified as having prevalent coronary heart disease (CHD), if they had either myocardial infarction or angina pectoris prior to recruitment. The prevalence of diabetes at baseline was assessed by using information on self-reported medical diagnosis, medication records and dieting behaviour. Physical activity was defined as the mean time spent on leisure time physical activities (hours/week) during summer and winter. Dietary habits, including alcohol consumption during the preceding year, were assessed by a validated self-administered food frequency questionnaire. All interviews and physical examinations were standardized and conducted by trained personnel [19].

### Statistical Analysis

Men had lower NT-proBNP levels than women; therefore, sex-specific tertiles were generated. Age- and sex-adjusted baseline characteristics were compared across NT-proBNP tertiles within the sub-cohort and between cases and the sub-cohort using analysis of covariance.

The association between NT-proBNP and the risk of HF was investigated by calculating hazard ratios (HR) and 95% confidence intervals (CI) across NT-proBNP tertiles using Cox proportional hazards regression modified by Prentice [25]. Age was used as the underlying time variable in the counting process with entry time defined as the participants' age at recruitment and exit time as age at time of HF diagnosis or censoring.

The baseline hazard function was allowed to vary by age (one-year integer). Three adjustment models were used to take into account a priori selected potential confounders: sex-adjusted (Model 1), additionally adjusted for educational degree (no vocational training/vocational training (reference); technical college; university), physical activity (hours/week), smoking status (never smoker (reference); former smoker; smoker <20cigarettes/day, smoker ≥20cigarettes/day), alcohol consumption (men: 0 g/day; >0–≤12 g/day (reference); >12–≤24 g/day; >24 g/day; women: 0 g/day; >0–≤6 g/day (reference); >6–≤12 g/day; >12 g/day), BMI and WC (continuously (if applicable)), prevalent diseases (diabetes, CHD, hypertension (present/absent)) (Model 2), and further adjusted for hsCRP, creatinine, total and HDL cholesterol (quartiles) (Model 3).

The shape of the associations between NT-proBNP and HF risk was determined using restricted cubic spline Cox regression analysis (Model 3) adapted to the case-cohort design with four knots at fixed values: 22.2, 30, 100, and 300 pg/ml. These reflected about the 15^th^, 25^th^, 75^th^ and 95^th^ percentiles of the NT-proBNP distribution in the entire case-cohort and guaranteed sufficient proportions of participants in all sections (including marginal). Furthermore, these values ensured that the number of participants did not differ greatly between knots. The respective median NT-proBNP value of 55 pg/ml was used as reference. Cox proportional hazards regression analysis per continuous increase of NT-proBNP was performed to demonstrate a linear association curve in direct comparison to the restricted cubic splines.

All analyses were performed overall and stratified for general obesity (BMI</≥30 kg/m^2^). Several sensitivity analyses were carried out, including the exclusion of non-“definite” HF cases and diabetics, and further adjustment for glucose and HbA1c. Additionally, stratified analyses were repeated interchanging BMI with parameters of abdominal obesity (WC and WHR).

The validity of the proportional hazards assumption was explored within the sub-cohort by calculating Schoenfeld residuals [26] for NT-proBNP and plotting them against time. No violation was observed. All statistical analyses were performed using SAS software package, release 9.2 (SAS Institute, Cary, NC) and p<0.05 was used to indicate statistical significance.

## Results

Analyses were based on a case-cohort comprising 1,150 non-cases and 210 cases of HF that occurred during a mean follow-up period of 8.3±1.5 years. The mean age of the sub-cohort was 50.5 years (standard deviation: 9.0 years). Medians and interquartile ranges of NT-proBNP (in pg/ml) were 35.4 [11–67.2] in men and 60.1 [39.2–97.0] in women of the sub-cohort and 84.8 [41.6–206.3] in male and 104.2 [54.7–203.5] in female HF cases. Table 1 depicts age- and sex-adjusted baseline characteristics of the study participants across NT-proBNP tertiles within the sub-cohort and compared between HF cases and the sub-cohort. The participants' age was higher with each higher tertile of NT-proBNP, whereas anthropometric parameters (BMI, WC and WHR) were lowest in the higher tertiles. BMI was inversely associated to NT-proBNP across tertiles (p = 0.02). Also, on a continuous scale a very weak negative correlation between BMI and NT-proBNP was observed in the sub-cohort (Spearman partial correlation coefficients adjusted for age and sex = −0.10). Comparing sub-cohort and HF cases, the latter were clearly older, more likely to be male and had higher average NT-proBNP concentrations. Furthermore, all anthropometric measures were higher in cases than in persons belonging to the sub-cohort.

**Table 1:** Age- and sex-adjusted baseline characteristics of the sub-cohort and the heart failure cases in EPIC-Potsdam.

	Sub-cohort^a^ (n = 1,163)				
	Tertiles of NT-proBNP^b^				
Characteristics	1st	2nd	3rd	Sub-cohort^a^	HF Cases^c^
	(n = 385)	(n = 390)	(n = 388)	(n = 1,163)	(n = 197)
**NT-proBNP** d, pg/ml					
Men	25 (26)	36 (24)	184 (25)	91 (15)	190 (29)
Women	31 (4)	63 (4)	148 (4)	82 (5)	222 (17)
**Socio-demographics**					
Age^e^, years	47.4 (8.3)	50.2 (9.1)	53.8 (8.5)	50.5 (9.0)	59.0 (6.4)
Women^e^, %	61.6	60.8	60.8	61.0	35.0
Educational degree, %					
(No) vocational training	38.8	38.4	37.2	38.3	41.4
Technical college	24.6	20.4	21.9	22.6	26.3
University	36.6	41.2	40.9	39.1	32.2
**Anthropometry**					
BMI^d^, kg/m^2^	26.6 (0.2)	26.1 (0.2)	25.8 (0.2)	26.3 (0.1)	28.0 (0.3)
Men	27.2 (0.3)	27.1 (0.3)	26.6 (0.3)	27.0 (0.2)	28.4 (0.3)
Women	26.1 (0.3)	25.4 (0.3)	25.2 (0.3)	25.7 (0.2)	28.3 (0.3)
Waist circumference^d^, cm					
Men	94.6 (0.8)	95.0 (0.8)	93.8 (0.8)	94.9 (0.5)	99.0 (0.9)
Women	81.8 (0.7)	79.8 (0.7)	79.8 (0.7)	80.9 (0.4)	86.4 (1.4)
Waist to hip ratio^d^					
Men	0.94 (0.01)	0.95 (0.00)	0.94 (0.01)	0.95 (0.00)	0.97 (0.01)
Women	0.80 (0.00)	0.79 (0.00)	0.79 (0.00)	0.80 (0.00)	0.81 (0.01)
**Lifestyle**					
Physical activity, h/wk	0.92 (0.09)	1.10 (0.09)	0.99 (0.09)	0.98 (0.05)	0.79 (0.12)
Alcoholic intake, g/d	15.5 (0.8)	15.6 (0.8)	15.8 (0.8)	15.6 (0.5)	13.8 (1.2)
Smoking, %					
Never	44.8	41.3	44.3	44.0	36.3
Former	34.6	36.9	35.7	35.6	34.9
<20 cigarettes/day	15.7	16.2	13.5	14.8	17.1
≥20 cigarettes/day	4.9	5.6	6.5	5.6	11.7
**Medical history, %**					
Diabetes mellitus	5.5	2.1	4.6	4.6	19.3
Hypertension	49.1	46.9	58.3	53.0	64.7
Hyperlipidemia^f^	29.9	27.7	30.4	30.7	42.6
Coronary heart disease	5.2	6.0	13.9	9.3	24.7
**Biomarkers, mg/dl**					
hsCRP	0.21 (0.02)	0.19 (0.02)	0.28 (0.02)	0.23 (0.01)	0.32 (0.03)
Creatinine	0.82 (0.01)	0.81 (0.01)	0.83 (0.01)	0.82 (0.01)	0.88 (0.02)
Total cholesterol	203 (1.8)	197 (1.8)	193 (1.8)	199 (1.1)	202 (2.7)
HDL cholesterol	48.5 (0.7)	50.0 (0.7)	50.7 (0.7)	49.6 (0.4)	46.7 (0.1)

Baseline characteristics are expresses as age- and sex-adjusted means (standard error) or percentages.

^a^ the sub-cohort included 13 heart failure cases.

^b^ tertiles of N-terminal pro brain natriuretic peptide (NT-proBNP) have been generated sex-specifically.

^c^ included only participants, who became cases after baseline and where not in the sub-cohort.

^d^ expressed as age-adjusted means (standard error).

^e^ expressed as unadjusted means (standard deviation) or percentages.

^f^ prevalent hyperlipidemia was defined by self-reporting of a confirmed diagnosis and/or the use of antihyperlipidemic drugs.

Among participants of the sub-cohort, 16.8% were general obese (BMI≥30 kg/m^2^). Baseline characteristics according to tertiles of NT-proBNP in obese (n = 195) and non-obese (n = 968) sub-cohort members are presented in Table 2. As expected, obese participants had much higher proportions of prevalent diseases compared to those who were not obese. As seen from Table 2, in particular the proportion of diabetics was about 3 times higher in obese than in non-obese participants, which equals a proportion of 10.9% obese diabetics in the first tertile of NT-proBNP.

**Table 2:** Age- and sex-adjusted baseline characteristics of the sub-cohort (n = 1,163) according to tertiles of NT-proBNP, stratified by status of obesity.

		Tertiles of NT-proBNP^a^		
	Characteristics	1st	2nd	3rd
**Non-obese (BMI<30)**		**(n = 324)**	**(n = 322)**	**(n = 322)**
	NT-proBNP^b^, pg/ml	26.0 (5.1)	53.3 (5.0)	154.3 (5.1)
	BMI, kg/m^2^	25.2 (0.1)	24.7 (0.1)	24.7 (0.1)
	Age^c^, years	47.1 (8.1)	49.0 (8.9)	53.3 (8.5)
	Women^c^, %	60.8	62.7	60.9
	Physical activity, h/wk	0.88 (0.1)	1.16 (0.1)	1.02 (0.1)
	Alcoholic intake, g/d	16.2 (0.8)	15.7 (0.8)	15.9 (0.9)
	Current smoking, %	20.1	21.7	21.4
	University degree, %	38.5	44.5	42.7
	Medical history, %			
	Diabetes mellitus	4.5	1.2	3.5
	Hypertension	43.8	40.1	54.2
	Hyperlipidemia^d^	27.7	24.7	26.3
	Coronary heart disease	5.2	4.7	13.1
**Obese (BMI≥30)**		**(n = 61)**	**(n = 68)**	**(n = 66)**
	NT-proBNP^b^, pg/ml	34.4 (57.7)	44.8 (52.7)	210.3 (53.6)
	BMI, kg/m^2^	33.1 (0.4)	33.2 (0.4)	32.7 (0.4)
	Age^c^, years	49.2 (9.3)	55.8 (7.8)	56.2 (7.9)
	Women^c^, %	65.6	51.5	60.6
	Physical activity, h/wk	1.20 (0.2)	0.78 (0.2)	0.81 (0.2)
	Alcoholic intake, g/d	11.8 (2.0)	15.0 (1.9)	15.4 (1.9)
	Current smoking, %	23.5	21.9	13.1
	University degree, %	28.9	24.7	29.7
	Medical history, %			
	Diabetes mellitus	10.9	5.8	11.3
	Hypertension	71.0	81.6	81.9
	Hyperlipidemia^d^	38.4	43.3	51.6
	Coronary heart disease	4.8	12.4	17.8

Baseline characteristics are expresses as age- and sex-adjusted means (standard error) or percentages.

^a^ tertiles of N-terminal pro brain natriuretic peptide (NT-proBNP) have been generated sex-specifically.

^b^ expressed as age-adjusted means (standard error).

^c^ expressed as unadjusted means (standard deviation) or percentages.

^d^ prevalent hyperlipidemia was defined by self-reporting of a confirmed diagnosis and/or the use of antihyperlipidemic drugs.

In Table 3 HRs of HF according to tertiles of NT-proBNP are shown overall and stratified for obesity status. Overall, persons within the third tertile of NT-proBNP had a 2- to 3-times higher risk of HF compared to participants from the first tertile. This result was irrespective of the analytic model applied. In stratified analyses, however, a clear linear trend was identified among non-obese participants while a U-shape was recognizable in obese individuals. These findings were again independent of the different multivariable adjustments applied in the three analytic models. In contrast, using the first tertile as reference category in obese, the respective HRs (95%CI) were: 0.30 (0.10–2.96) for the second and 1.14 (0.41–3.17) for the third tertile after multivariable adjustment.

**Table 3:** Association between NT-proBNP and the risk of incident heart failure, overall and stratified by status of obesity.

	Tertiles of NT-proBNP			
	1st	2nd	3rd	
**Non-cases/cases n**	**385/27**	**386/44**	**379/139**	
Person-years^a^	3,221	3,233	3,146	
	**HR**	**HR (95% CI)**	**HR (95% CI)**	**p** *
Model 1^b^	Reference	1.12 (0.65–1.92)	2.57 (1.59–4.17)	<0.01
Model 2^c^ #	Reference	1.11 (0.62–2.00)	2.49 (1.48–4.21)	<0.01
Model 3^d^ #	Reference	1.17 (0.64–2.12)	2.56 (1.49–4.41)	<0.01
**Non-obese (BMI<30)**				
**Non-cases/cases n**	**324/15**	**321/29**	**316/90**	
Person-years^a^	2,720	2,684	2,641	
	**HR**	**HR (95% CI)**	**HR (95% CI)**	**p** *
Model 1^b^	Reference	1.48 (0.74–2.95)	2.99 (1.61–5.54)	<0.01
Model 2^c^	Reference	1.56 (0.79–3.07)	2.64 (1.40–4.98)	<0.01
Model 3^d^	Reference	1.72 (0.85–3.49)	2.72 (1.42–5.22)	<0.01
**Obese (BMI**≥**30)**				
**Non-cases/cases n**	**61/12**	**65/15**	**63/49**	
Person-years^a^	501	549	504	
	**HR**	**HR (95% CI)**	**HR (95% CI)**	**p** $
Model 1^b^	1.92 (0.71–5.18)	Reference	3.30 (1.59–6.84)	<0.01
Model 2^c^	3.58 (1.13–11.35)	Reference	3.95 (1.69–9.23)	<0.01
Model 3^d^	3.29 (1.04–10.40)	Reference	3.74 (1.52–9.21)	<0.01

^a^ Person years are calculated from the sub-cohort (n = 1,163) only.

^b^ adjusted for sex, stratified for baseline age.

^c^ Model 1 further adjusted for educational degree, physical activity, smoking status, alcohol consumption, waist circumference, and prevalent diseases (diabetes, coronary heart disease, hypertension).

^d^ Model 2 further adjusted for biomarkers (hsCRP, creatinine, total cholesterol and HDL cholesterol).

*p for trend across tertiles was calculated by using tertiles of N-terminal pro brain natriuretic peptide (NT-proBNP) as a categorical variable in the respective Cox proportional hazards regression models.

^$^the p value for nonlinearity was calculated by Wald chisquare test following restricted cubic spline Cox regression analysis.

^#^in the non-stratified models, body mass index (BMI) was also included in the adjustment set.

**Figure 1:**
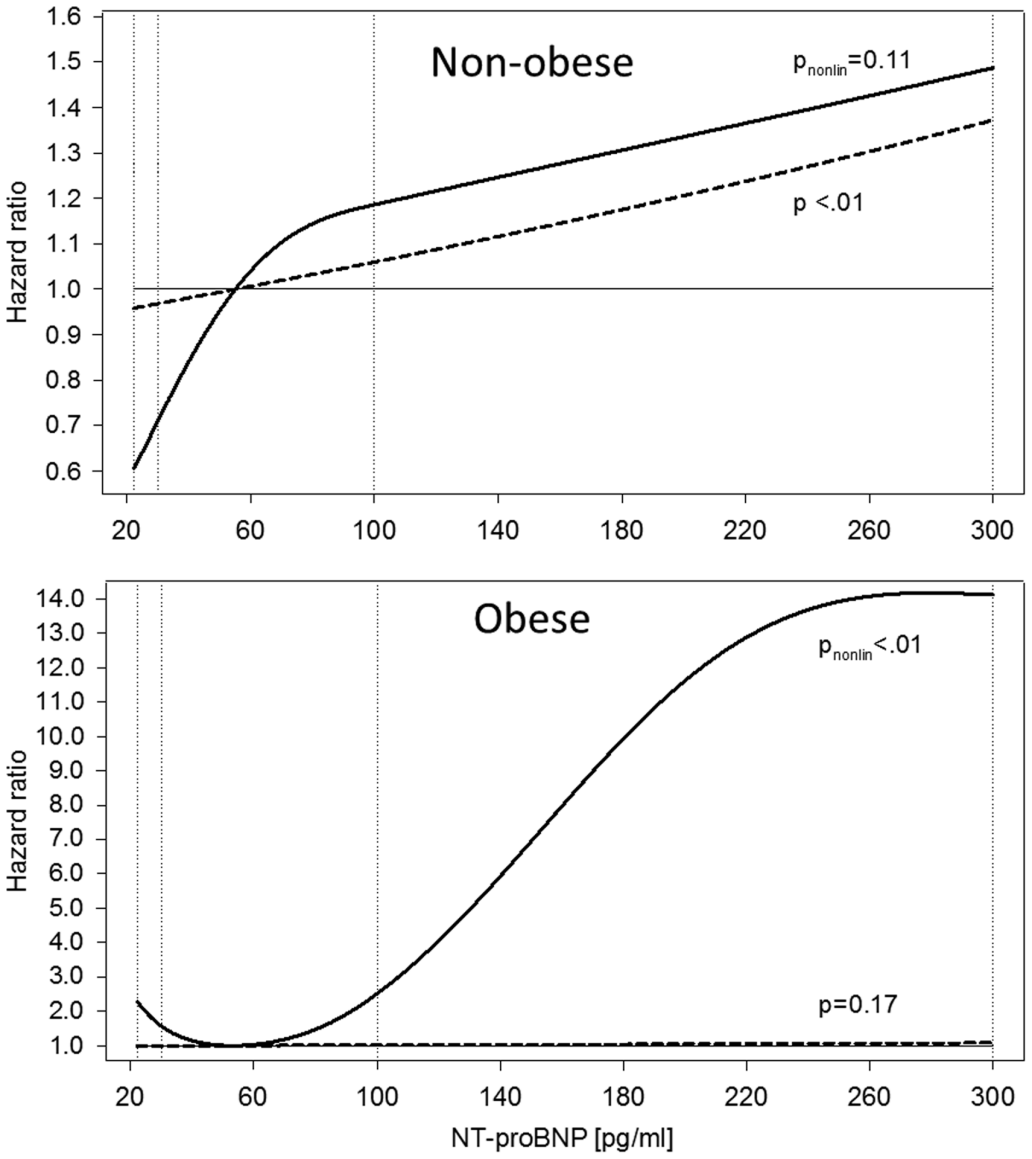
Hazard ratio curves for the association between NT-proBNP levels and the risk of heart failure in non-obese (n = 1,095) and obese (n = 265) participants. Results are illustrated for non-obese (BMI<30 kg/m^2^) and obese (BMI≥30 kg/m^2^) participants. The solid lines indicate hazard ratios of heart failure as obtained by restricted cubic spline Cox regression with knots (marked by vertical dotted lines) placed at fixed values (∼15th, 25th, 75th and 95th percentile of the distribution of NT-proBNP in the entire case-cohort). The reference was set at ∼50th percentile = 55 pg/ml of NT-proBNP. The dashed lines indicate hazard ratios of heart failure as observed by Cox regression analysis per continuous increment of NT-proBNP. Hazard ratios curves are adjusted for age, sex, educational degree, physical activity, smoking status, alcohol consumption, WC, prevalent diseases (diabetes, CHD, hypertension) and biomarkers (hsCRP, creatinine, total cholesterol and HDL cholesterol).

Results from the restricted cubic spline Cox regression supported these findings (Figure 1). HRs are plotted comparing a linear (dashed line) and a non-linear (solid line) relationship between NT-proBNP and the risk of HF for non-obese and obese participants. In non-obese, the association was approximately linear, while in obese the non-linear model fitted the data better than the linear model. Actually, in the linear model no effect was visible, as the risk-increase with both very low and very high values of NT-proBNP and the low risk with intermediate NT-proBNP values canceled each other out.

The relationship between NT-proBNP and HF risk was also modified by abdominal obesity as defined by WC or WHR (Tables S1, S2, S3, S4). While results from analyses on WC (Table S3) were comparable to those of BMI, the U-shaped association between NT-proBNP and HF risk was less pronounced in obese defined by WHR. In particular, the higher HF risk originally seen in those with low NT-proBNP levels became weaker (Table S4).

Among obese participants, both the exclusion of diabetics (n = 44) and of non-definite HF cases (n = 20) even strengthened the U-shaped association, although lowering the precision of the estimate (HRs (95% CI) for obese participants after exclusion of diabetics: tertile 1: 6.81 (2.21–21.03), tertile 2: reference, tertile 3: 7.28 (2.69–19.68), and after exclusion of non-definite cases: tertile 1: 3.42 (0.85–13.84), tertile 2: reference, tertile 3: 4.16 (1.44–12.02)). Also further adjustment for glucose and HbA1c did not substantially alter the risk estimates in both obese and non-obese.

## Discussion

In this prospective study, NT-proBNP was positively associated with the risk of HF. However, in stratified analyses differences were observed between non-obese and obese participants. In the non-obese, the HF risk rose monotonously with higher NT-proBNP concentration. In contrast, a U-shaped relationship between NT-proBNP and HF risk was observed in obese. Taking into account the shape of the relationship, it could be demonstrated that those with both low and high NT-proBNP concentrations were at higher risk of HF.

Evidence on the association between NT-proBNP and the future HF risk is limited to four cohort studies: the Malmö Diet and Cancer Study [8], the Rotterdam Study [9], the Rochester Epidemiology Project [10] and the Cardiovascular Health Study [11], which were conducted mainly in elderly adults. These studies consistently reported positive associations between NT-proBNP and HF risk and therefore confirm the overall, non-stratified result of the present analysis. However, this study went further and it presents for the first time analyses that examined the impact of obesity on the association between NT-proBNP and HF risk in a prospective manner. The influence of obesity on NT-proBNP has to a great extend been investigated by cross-sectional studies, e.g. Rivera et al. and Cheng et al. [17], [18], but did not include the relation to HF incidence. However, some clinical longitudinal studies [15], [16], [27]–[29] have investigated the risk of mortality according to NT-proBNP concentrations in different weight classes. Those reported inconsistent results but largely confirmed the suitability of NT-proBNP as prognostic marker for mortality in both obese and non-obese cardiac patients. However, Christensen et al. [15] proposed the use of BMI-specific NT-proBNP cut-points to optimize sensitivity in obese individuals with possible decompensated HF.

Different pathophysiological mechanisms might be responsible for a higher risk of HF with low NT-proBNP in obese individuals. First, BNP secretion may be reduced in some obese with high risk of HF. One biologically plausible explanation is the O-glycolysation of proBNP. If proBNP is present in glycosylated form, especially near the cleavage site, it cannot be further converted into BNP and NT-proBNP [30]. This glycosylation process might be promoted by elevated blood glucose levels and indicate an initial or advanced state of insulin resistance. Indeed, insulin has also been linked to natriuretic peptides [31], [32]. In particular, Khan and colleagues [31] observed markedly lower NT-proBNP levels in both obese and non-obese, insulin resistant participants compared to non-obese, insulin sensitive ones. When comparing non-obese with obese insulin sensitive participants, differences in NT-proBNP levels were negligible. This might also explain the higher proportion of prevalent diabetics among non-obese participants with low NT-proBNP levels observed in the present study. Second, composition and type of adipose tissue could have an influence on the degradation of NT-proBNP. Pivovarova et al. [32] studied the gene expression of natriuretic peptide clearance receptors in visceral and subcutaneous adipose tissue depots and the acute effect of insulin infusion. They observed higher gene expression in visceral adipose tissue compared to subcutaneous adipose tissue, and the latter being up-regulated by insulin. Indeed, both subcutaneous and visceral adipose tissues have been associated with lower NT-proBNP [18], suggesting a more favorable adipose tissue distribution with higher NT-proBNP levels. Interestingly, analyses with parameters of abdominal obesity differed only with regard to WHR, where the U-shape was less pronounced in obese. In fact, WHR has been considered advantageous over BMI for predicting CVD and the metabolic syndrome [33], [34]. WHR might contain information that improves discrimination between obese and non-obese participants.

Some limitations merit consideration: First, the study was limited by the relatively small number of obese participants and thereby the low number of cases in the stratified analysis. Therefore, the risk of chance findings was increased, and the authors refrained from several more detailed analyses. Second, the risk of residual and unmeasured confounding cannot be ruled out. The potential of confounding may be higher in obese compared to non-obese, demonstrated by greater differences in baseline characteristics and hence in risk estimates before and after multivariable adjustment. Despite a very comprehensive consideration of possible confounders, factors such as insulin resistance could not be accounted for in the present investigation. However, in addition to the adjustment for diabetes, further adjustment for glucose and HbA1c did not change the U-shaped association observed in obese participants and, on the contrary, the exclusion of diabetics even strengthened this finding. Nevertheless, there might be a considerable difference between impaired fasting glucose and impaired glucose tolerance in the onset of heart failure, which could not be accounted for [35].

Strengths of the present study include its prospective design and the wide range of considered covariates and exposure variables, including measures of general and abdominal obesity. Furthermore, analyses were performed using NT-proBNP rather than BNP, which might be of beneficial value: NT-proBNP is a biologically inert molecule, reported to be very stable, assuming a lower amount of degradation after years of storage. Lastly, case assessment was carried out carefully and it was distinguished between definite, probable and possible cases according to the ESC criteria. Excluding probable and possible cases from the analysis even strengthened the observed associations.

## Conclusions

The findings of the present analyses demonstrate that, regardless of obesity status, high circulating concentrations of NT-proBNP were positively associated to incident HF. These findings are of great importance as they confirm the clinical feasibility of NT-proBNP in obese individuals, who still represent a crucial risk group, where sensitivity and specificity of traditional devices might be limited [13]. However, the present analysis suggests that people with obesity and low plasma NT-proBNP are also at higher risk of HF. Further studies to support these findings are needed.

## Supporting Information

Figure S1 **Flow chart of the number and reasons of excluded participants in the case-cohort design.** * including 13 cases of heart failure (HF)
(TIF)

Table S1 **Age- and sex-adjusted baseline characteristics of the sub-cohort (n = 1,163), according to tertiles of NT-proBNP, stratified by status of obesity defined by waist circumference (WC).**
(DOC)

Table S2 **Age- and sex-adjusted baseline characteristics of the sub-cohort (n = 1,163), according to tertiles of NT-proBNP, stratified by status of obesity defined by waist to hip ratio (WHR).**
(DOC)

Table S3 **Association between NT-proBNP and the risk of incident heart failure, stratified by status of obesity defined by waist circumference (WC).**
(DOC)

Table S4 **Association between NT-proBNP and the risk of incident heart failure, stratified by status of obesity defined by waist to hip ratio (WHR).**
(DOC)
