# Supplementary figures and images for: Relationship between N-Terminal Pro-Brain Natriuretic Peptide, Obesity and the Risk of Heart Failure in Middle-Aged German Adults
Source: PLoS One. 2014 Nov 25;9(11):e113710. doi: 10.1371/journal.pone.0113710 (PMC4244121; doi:10.1371/journal.pone.0113710)

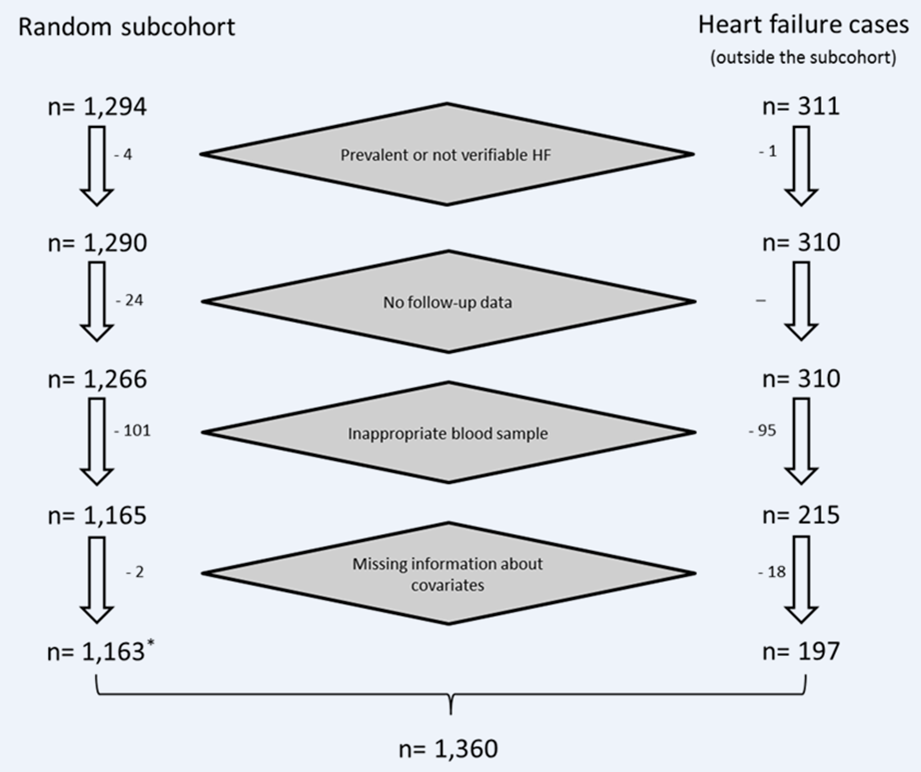

Supplement: Figure S1 — Flow chart of the number and reasons of excluded participants in the case-cohort design. * including 13 cases of heart failure (HF) (TIF) [file pone.0113710.s001.tif]
