# Supplementary material for: Relationship between N-Terminal Pro-Brain Natriuretic Peptide, Obesity and the Risk of Heart Failure in Middle-Aged German Adults
Source: PLoS One. 2014 Nov 25;9(11):e113710. doi: 10.1371/journal.pone.0113710 (PMC4244121; doi:10.1371/journal.pone.0113710)
Supplement: Table S1 — Age- and sex-adjusted baseline characteristics of the sub-cohort (n = 1,163), according to tertiles of NT-proBNP, stratified by status of obesity defined by waist circumference (WC). (DOC) [file pone.0113710.s002.doc]

Table S1 Age- and sex-adjusted baseline characteristics of the sub-cohort (n=1,163), according to tertiles of NT-proBNP, stratified by status of obesity defined by waist circumference (WC)

|  |  | **Tertiles of NT-proBNP**^a^ | | |
| --- | --- | --- | --- | --- |
|  | **Characteristics** | **1st** | **2nd** | **3rd** |
| **Non-obese** | **(WC: m <102cm, w <88cm)** | **(n=299)** | **(n=293)** | **(n=286)** |
|  | NT-proBNP^b^, pg/ml | 25.0 (4.6) | 52.5 (4.6) | 146.8 (4.8) |
|  | Age^c^, years | 46.5 (8.1) | 48.6 (8.7) | 52.4 (8.6) |
|  | Women^c^, % | 58.5 | 61.4 | 60.1 |
|  | Physical activity, h/wk | 0.94 (0.1) | 1.22 (0.1) | 1.06 (0.1) |
|  | Alcoholic intake, g/d | 16.1 (0.8) | 15.1 (0.8) | 15.3 (0.9) |
|  | Current smoking, % | 20.4 | 22.5 | 21.7 |
|  | University degree, % | 38.9 | 45.4 | 42.8 |
|  | Medical history, % |  |  |  |
|  | Diabetes mellitus | 3.0 | 1.2 | 1.8 |
|  | Hypertension | 40.0 | 37.4 | 52.6 |
|  | Hyperlipidemia^d^ | 27.6 | 22.2 | 25.6 |
|  | Coronary heart disease | 3.5 | 4.5 | 11.4 |
| **Obese** | **(WC: m ≥102cm, w ≥88cm)** | **(n=86)** | **(n=97)** | **(n=102)** |
|  | NT-proBNP^b^, pg/ml | 31.8 (41.1) | 51.0 (37.1) | 213.3 (37.1) |
|  | Age^c^, years | 50.4 (8.4) | 55.2 (8.3) | 57.7 (7.0) |
|  | Women^c^, % | 72.1 | 58.8 | 62.7 |
|  | Physical activity, h/wk | 0.92 (0.2) | 0.74 (0.2) | 0.72 (0.2) |
|  | Alcoholic intake, g/d | 13.4 (1.9) | 17.2 (1.7) | 17.4 (1.7) |
|  | Current smoking, % | 22.4 | 19.4 | 15.1 |
|  | University degree, % | 31.1 | 28.7 | 33.3 |
|  | Medical history, % |  |  |  |
|  | Diabetes mellitus | 14.0 | 4.7 | 14.2 |
|  | Hypertension | 75.5 | 76.5 | 80.8 |
|  | Hyperlipidemia^d^ | 35.3 | 45.0 | 46.7 |
|  | Coronary heart disease | 10.6 | 10.5 | 22.2 |

Baseline characteristics are expresses as age- and sex-adjusted means (standard error) or percentages

^a^ tertiles of N-terminal pro brain natriuretic peptide (NT-proBNP) have been generated sex-specifically

^b^ expressed as age-adjusted means (standard error)

^c^ expressed as unadjusted means (standard deviation) or percentages

^d^ prevalent hyperlipidemia was defined by self-reporting of a confirmed diagnosis and/or the use of antihyperlipidemic drugs.
