# Supplementary material for: Relationship between N-Terminal Pro-Brain Natriuretic Peptide, Obesity and the Risk of Heart Failure in Middle-Aged German Adults
Source: PLoS One. 2014 Nov 25;9(11):e113710. doi: 10.1371/journal.pone.0113710 (PMC4244121; doi:10.1371/journal.pone.0113710)
Supplement: Table S2 — Age- and sex-adjusted baseline characteristics of the sub-cohort (n = 1,163), according to tertiles of NT-proBNP, stratified by status of obesity defined by waist to hip ratio (WHR). (DOC) [file pone.0113710.s003.doc]

**Table S2 Age- and sex-adjusted baseline characteristics of the sub-cohort (n=1,163), according to tertiles of NT-proBNP, stratified by status of obesity defined by waist to hip ratio (WHR)**

|  |  | **Tertiles of NT-proBNP**^a^ | | |
| --- | --- | --- | --- | --- |
|  | **Characteristics** | **1st** | **2nd** | **3rd** |
| **Non-obese** | **(WHR: m ≤1.0, w ≤0.85)** | **(n=321)** | **(n=314)** | **(n=310)** |
|  | NT-proBNP^b^, pg/ml | 26.8 (11.7) | 53.0 (11.7) | 159.7 (12.1) |
|  | Age^c^, years | 46.7 (8.1) | 48.7 (8.9) | 52.9 (8.6) |
|  | Women^c^, % | 59.2 | 60.2 | 60.3 |
|  | Physical activity, h/wk | 0.95 (0.1) | 1.13 (0.1) | 1.09 (0.1) |
|  | Alcoholic intake, g/d | 15.7 (0.8) | 15.1 (0.8) | 15.0 (0.8) |
|  | Current smoking, % | 20.1 | 20.0 | 19.4 |
|  | University degree, % | 38.4 | 43.6 | 43.2 |
|  | Medical history, % |  |  |  |
|  | Diabetes mellitus | 3.2 | 1.1 | 2.5 |
|  | Hypertension | 43.8 | 39.3 | 54.5 |
|  | Hyperlipidemia^d^ | 26.8 | 22.2 | 28.8 |
|  | Coronary heart disease | 3.8 | 4.5 | 12.3 |
| **Obese** | **(WHR: m >1.0, w >0.85)** | **(n=64)** | **(n=76)** | **(n=78)** |
|  | NT-proBNP^b^, pg/ml | 28.5 (15.5) | 50.6 (13.8) | 178.4 (13.7) |
|  | Age^c^, years | 51.2 (8.5) | 56.4 (7.0) | 57.3 (7.3) |
|  | Women^c^, % | 73.4 | 63.2 | 62.8 |
|  | Physical activity, h/wk | 0.82 (0.2) | 0.99 (0.2) | 0.53 (0.2) |
|  | Alcoholic intake, g/d | 14.3 (2.2) | 18.3 (2.0) | 19.3 (2.0) |
|  | Current smoking, % | 21.2 | 28.8 | 24.6 |
|  | University degree, % | 29.0 | 31.7 | 29.1 |
|  | Medical history, % |  |  |  |
|  | Diabetes mellitus | 16.9 | 5.1 | 15.4 |
|  | Hypertension | 69.6 | 79.2 | 80.0 |
|  | Hyperlipidemia^d^ | 42.6 | 51.0 | 40.9 |
|  | Coronary heart disease | 12.1 | 11.7 | 22.0 |

Baseline characteristics are expresses as age- and sex-adjusted means (standard error) or percentages

^a^ tertiles of N-terminal pro brain natriuretic peptide (NT-proBNP) have been generated sex-specifically

^b^ expressed as age-adjusted means (standard error)

^c^ expressed as unadjusted means (standard deviation) or percentages

^d^ prevalent hyperlipidemia was defined by self-reporting of a confirmed diagnosis and/or the use of antihyperlipidemic drugs.
