# Supplementary material for: Relationship between N-Terminal Pro-Brain Natriuretic Peptide, Obesity and the Risk of Heart Failure in Middle-Aged German Adults
Source: PLoS One. 2014 Nov 25;9(11):e113710. doi: 10.1371/journal.pone.0113710 (PMC4244121; doi:10.1371/journal.pone.0113710)
Supplement: Table S3 — Association between NT-proBNP and the risk of incident heart failure, stratified by status of obesity defined by waist circumference (WC). (DOC) [file pone.0113710.s004.doc]

## Table S3 Association between NT-proBNP and the risk of incident heart failure, stratified by status of obesity defined by waist circumference (WC)

|  | **Tertiles of NT-proBNP** | | |
| --- | --- | --- | --- |
|
|  | **1st** | **2nd** | **3rd** |
| **Non-obese** |  |  |  |
| **(WC: m <102cm, w <88cm)** |  |  |  |
| **Non-cases / cases n** | **299 / 8** | **292 / 26** | **282 / 76** |
| Person-years^a^ | 2,512 | 2,453 | 2,357 |
|  | **HR** | **HR (95% CI)** | **HR (95% CI)** |
| Model 1^b^ | Reference | 2.55 (1.11-5.85) | 4.74 (2.22-10.09) |
| Model 2^c^ | Reference | 2.58 (1.06-6.24) | 3.71 (1.64-8.43) |
|  |  |  |  |
| **Obese** |  |  |  |
| **(WC: m ≥102cm, w ≥88cm)** |  |  |  |
| **Non-cases / cases n** | **86 / 19** | **94 / 18** | **97 / 63** |
| Person-years^a^ | 709 | 780 | 789 |
|  | **HR** | **HR (95% CI)** | **HR (95% CI)** |
| Model 1^b^ | 2.25 (1.01-5.01) | Reference | 3.32 (1.78-6.21) |
| Model 2^c^ | 2.88 (1.10-7.51) | Reference | 3.81 (1.72-8.44) |

^a^ Person years are calculated from the sub-cohort (n=1,163) only

^b^ adjusted for sex, stratified for baseline age

^c^ Model 1 further adjusted for educational degree, physical activity, smoking status, alcohol consumption, body mass index, prevalent diseases (diabetes, coronary heart disease, hypertension) and biomarkers ( hsCRP, creatinine, total cholesterol and HDL cholesterol)
