# Supplementary material for: Relationship between N-Terminal Pro-Brain Natriuretic Peptide, Obesity and the Risk of Heart Failure in Middle-Aged German Adults
Source: PLoS One. 2014 Nov 25;9(11):e113710. doi: 10.1371/journal.pone.0113710 (PMC4244121; doi:10.1371/journal.pone.0113710)
Supplement: Table S4 — Association between NT-proBNP and the risk of incident heart failure, stratified by status of obesity defined by waist to hip ratio (WHR). (DOC) [file pone.0113710.s005.doc]

## Table S4 Association between NT-proBNP and the risk of incident heart failure, stratified by status of obesity defined by waist to hip ratio (WHR)

|  | **Tertiles of NT-proBNP** | | |
| --- | --- | --- | --- |
|
|  | **1st** | **2nd** | **3rd** |
| **Non-obese** |  |  |  |
| **(WHR: m ≤ 1.0, w ≤0.85)** |  |  |  |
| **Non-cases / cases n** | **321 / 17** | **313 / 26** | **304 / 86** |
| Person-years^a^ | 2,689 | 2,610 | 2,545 |
|  | **HR** | **HR (95% CI)** | **HR (95% CI)** |
| Model 1^b^ | Reference | 1.14 (0.58-2.22) | 2.51 (1.39-4.55) |
| Model 2^c^ | Reference | 1.28 (0.62-2.64) | 2.38 (1.22-4.64) |
|  |  |  |  |
| **Obese** |  |  |  |
| **(WHR: m >1.0, w >0.85)** |  |  |  |
| **Non-cases / cases n** | **64 / 10** | **73 / 18** | **75 / 53** |
| Person-years^a^ | 533 | 623 | 600 |
|  | **HR** | **HR (95% CI)** | **HR (95% CI)** |
| Model 1^b^ | 1.06 (0.42-2.69) | Reference | 3.01 (1.60-5.64) |
| Model 2^c^ | 1.27 (0.35-4.59) | Reference | 4.42 (0.98-12.29) |

^a^ Person years are calculated from the sub-cohort (n=1,163) only

^b^ adjusted for sex, stratified for baseline age

^c^ Model 1 further adjusted for educational degree, physical activity, smoking status, alcohol consumption, body mass index, prevalent diseases (diabetes, coronary heart disease, hypertension) and biomarkers ( hsCRP, creatinine, total cholesterol and HDL cholesterol)
